# Knockdown of METTL16 disrupts learning and memory by reducing the stability of MAT2A mRNA

**DOI:** 10.1038/s41420-022-01220-0

**Published:** 2022-10-28

**Authors:** Runjiao Zhang, Yizhou Zhang, Fangzhen Guo, Guannan Huang, Yan Zhao, Bingyu Chen, Chang Wang, Chengran Cui, Yichun Shi, Sha Li, Huixian Cui

**Affiliations:** 1 Department of Anatomy, Hebei Medical University, 050017 Shijiazhuang, Hebei China; 2 Neuroscience Research Center, Hebei Medical University, 050017 Shijiazhuang, Hebei China; 3 Hebei Key Laboratory of Neurodegenerative Disease Mechanism, 050017 Shijiazhuang, Hebei China; 4 Department of Ultrasound, The Second Hospital of Hebei Medical University, 050000 Shijiazhuang, Hebei China; 5 School of Nursing, Hebei Medical University, 050017 Shijiazhuang, Hebei China; 6 Grade 2019, Basic Medicine, Hebei Medical University, 050017 Shijiazhuang, Hebei China

**Keywords:** Cellular neuroscience, Hippocampus

## Abstract

N6-methyladenosine (m^6^A) is abundant in the mammalian brain and is considered to have a wide range of effects on learning and memory. Here, we found that the upregulated methyltransferase-like protein 16 (METTL16) in the hippocampal tissues of Morris water maze (MWM)-trained mice contributed to improved memory formation and hippocampal synaptic plasticity. Mechanismly, METTL16 promoted the expression of methionine adenosyltransferase 2A (MAT2A) by the m^6^A methylation of the MAT2A mRNA-3′UTR-end to increase its stability, and this involved in improving hippocampal global m^6^A levels, plasticity of dendritic spine, learning and memory. This study provides a new perspective to explore the regulatory mechanisms of m^6^A for learning and memory.

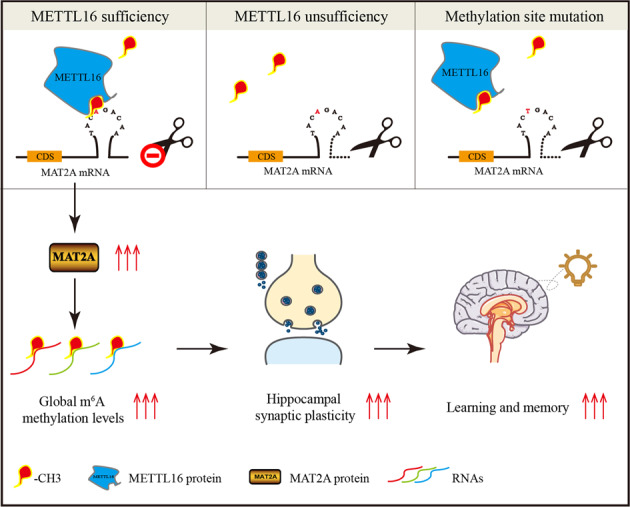

## Introcuction

Learning and memory processes can be widely considered as a synaptic process that needs local variation in synapse to cause stable changes in brain function [1]. The disruption of hippocampal synaptic plasticity is considered the cause of learning and memory impairment in age-related neurodegenerative diseases, including Alzheimer’s and Parkinson’s diseases [2–4]. It is worth noting that memory storage is subject to multiple layers of control, from post-translational modification at synapse to the production of RNA in nucleus [5–7]. The stability and translation of RNA transcripts have been shown to depend on N6-methyladenosine (m^6^A) modification [8, 9].

RNA m^6^A methylation is one of the most prominent and abundant forms of RNA modification in eukaryotic cells [10]. It is bidirectionally regulated by methyltransferases and demethylases (also called ‘writers’ and ‘erasers’, respectively), which work with binding proteins (also called ‘readers’) to regulate RNA fate [11]. In mammals, m^6^A methylation modification is widely distributed in many tissues, particularly in the brain [12]. Several recent reports focused on investigating the functional significance of m^6^A regulation in de novo RNA transcripts, including nuclear splicing, stability, translation, and subcellular localisation [13, 14], implying that m^6^A serves as a regulator to precisely fine-tune several physiological and pathophysiological processes over time in the brain [15]. The abundance of m^6^A and its emerging role as an important post-transcriptional regulator in the mammalian brain has gained considerable attention in the field of neuroepigenetics [16, 17].

In this study, we identified m^6^A-related proteins that play a key role in learning and memory. We used the Morris water maze (MWM) method to conduct learning and memory training in mice and collected the hippocampal tissues of the mice for proteomic data analysis. Then, the upregulated m^6^A methyltransferase methyltransferase-like 16 (METTL16) and consequent upregulated overall levels of m^6^A caught our attention, as upregulated m^6^A levels is considered to promote the transcriptome response to synaptic plasticity [18, 19]. Our study is the first to propose the role of METTL16 in memory formation and hippocampal synaptic plasticity. The mechanism of action of METTL16 was considered to promote the expression of methionine adenosyltransferase 2 A (MAT2A) by the m^6^A methylation of the MAT2A mRNA-3′UTR-end to increase its stability. MAT2A is a key enzyme for methyl donor synthesis [20–24]. Finally, our findings reveal a new m^6^A-based regulatory mechanism for learning and memory and provide novel insights into the potential mechanisms involved in the improvement of learning and memory deficits.

## Results

### METTL16 is upregulated in the hippocampi of MWM-trained mice

**Fig. 1:**
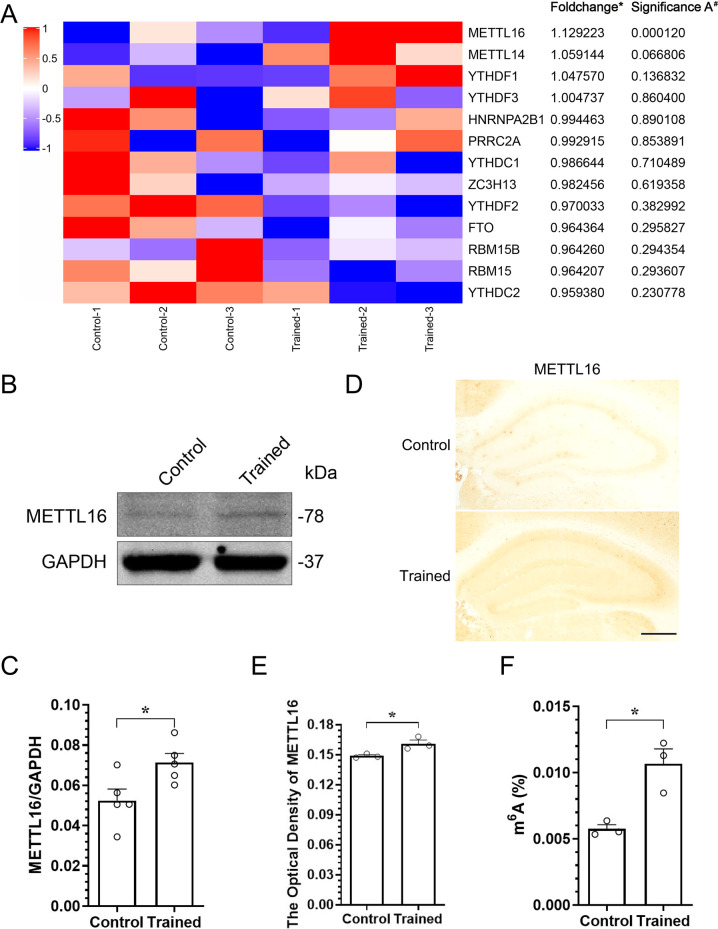
METTL16 is upregulated in the hippocampi of MWM-trained mice. **A** Expression levels of RNA m^6^A methylation modification proteins in the hippocampi of the MWM training mice from our proteomic data (PXD032022; http://proteomecentral.proteomexchange.org). *The fold change of trained group (*n* = 3) vs control group (*n* = 3). ^#^MaxQuant was used to assess the statistical significance [28]. **B**, **C** Representative western blotting (**B**) and quantification of METTL16 (**C**) in the hippocampi of MWM-trained (*n* = 5) and untrained mice (*n* = 5). **D**, **E** Representative IHC images (**D**) and quantification of METTL16 (**E**) in the hippocampi of MWM-trained (*n* = 3) and untrained mice (*n* = 3); Scale bars = 500 μm. **F** Overall levels of m^6^A in the hippocampi of MWM-trained (*n* = 3) and untrained mice (*n* = 3) tested by m^6^A colorimetry. Data shown as the mean ± standard error of the mean (SEM). *P-*values were determined by two-tailed *t*-test. *P < 0.05.

An increasing number of studies have demonstrated that m^6^A methylation plays an important role in learning and memory [25, 26]. To identify m^6^A-related proteins that play a key role in learning and memory, we conducted MWM training in mice and collected hippocampal tissues for proteomic data analysis. Proteomic data were deposited in the ProteomeXchange Consortium (http://proteomecentral.proteomexchange.org) via the iProX partner repository [27] with the dataset identifier PXD032022. The results showed that METTL16 was the most upregulated m^6^A methylation modification proteins in the hippocampi of MWM-trained mice compared to that in the untrained group (fold change = 1.129223, significance A [28] = 0.000120) (Fig. 1A). Subsequently, western blotting and immunohistochemical (IHC) staining validated that the expression level of METTL16 was upregulated in the hippocampi of MWM-trained mice compared to that in the control group (Fig. 1B–E). Further, m^6^A colourimetric quantification revealed that this upregulation was accompanied by an increase in the overall levels of m^6^A (Fig. 1F), which is considered to be closely related to learning and memory formation by promoting the transcriptome response to synaptic plasticity [18, 19]. Therefore, it is reasonable to speculate that METTL16 may contribute to the enhancement of learning and memory.

### Knockdown of METTL16 in the hippocampi causes learning and memory deficit

**Fig. 2:**
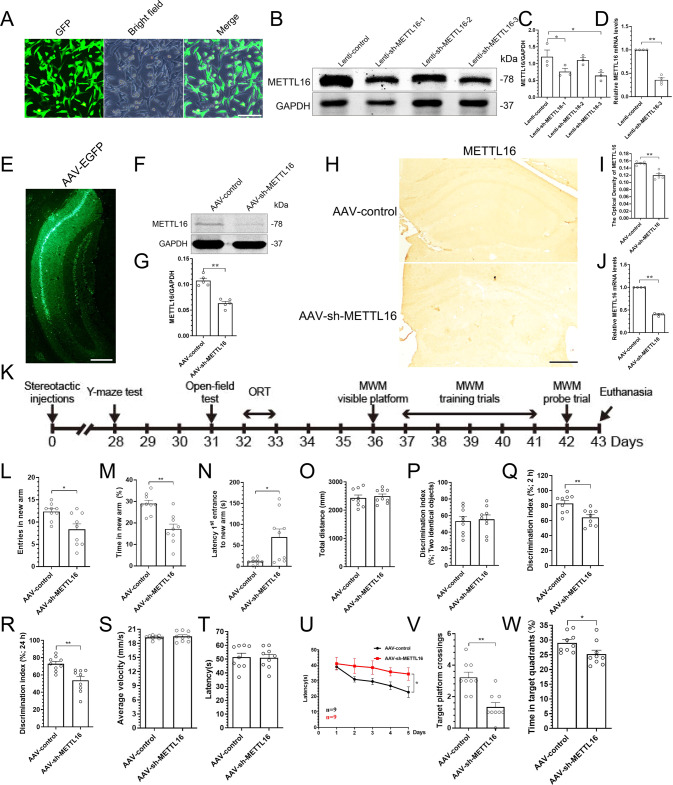
Knockdown of METTL16 in the hippocampi causes learning and memory deficit. **A** Representative fluorescence images of HT22 cells after puromycin was used to kill the cells not transfected with lentivirus; Scale bars = 100 μm. **B**–**D** Knockdown efficiency of METTL16 in HT22 cells was detected by western blotting (*n* = 3) (**B**, **C**) and qRT–PCR (*n* = 4) (**D**). **E** Representative image of stereotactic injections of AAV-sh-METTL16 in the hippocampi; Scale bars = 500 μm. **F**, **G** Representative western blotting (**F**) and quantification of METTL16 (**G**) in the hippocampi of METTL16 knockdown (*n* = 5). **H**, **I** Representative IHC images (**H**) and quantification of METTL16 (**I**) in the hippocampi of METTL16 knockdown (*n* = 5); Scale bars = 500 μm. **J** Knockdown efficiency of METTL16 in the hippocampi was detected by qRT–PCR (*n* = 4). **K** Schematic diagram of the experimental design. Y-maze test was initiated at Day 28 of stereotactic. After 2 days of relaxation, open-field test was employed at Day 31, followed by ORT experiments for 2 days. After 2 days of relaxation, three phases of WMW (visible platform phase, training trials phase, and probe trial phase) were performed in sequence for 7 days. Finally, hippocampi were collected at Day 43 of stereotactic injections for subsequent in vitro experiments. **L**–**O** Entries in new arm (**L**), time in new arm (**M**), latency 1st entrance to new arm (**N**), and total distance (**O**) of mice in the AAV-control (*n* = 9) and AAV-sh-METTL16 group (*n* = 9) were tested in the Y-maze test. **P**–**R** Discrimination index (DI) for new objects of mice in the AAV-control (*n* = 9) and AAV-sh-METTL16 group (*n* = 9) was tested in 2 h (**Q**) and 24 h (**R**) after excluding the mice’s preference for object location (**P**) in the ORT experiment. **S**, **T** Average velocity (**S**) and latency to find the platform (**T**) of mice in the AAV-control (*n* = 9) and AAV-sh-METTL16 group (*n* = 9) were explored in the visible platform phase of MWM. **U** Latency to find the platform of mice in the AAV-control (*n* = 9) and AAV-sh-METTL16 group (*n* = 9) were explored in the training trials phase of MWM. **V**, **W** Number of platform crossings (**V**) and time in target quadrants (**W**) of mice in the AAV-control (*n* = 9) and AAV-sh-METTL16 group (*n* = 9) were employed in the probe trial phase of MWM. Data shown as the mean ± SEM. *P-*values were determined by one-way ANOVA (**C**), two-tailed *t*-test (**D**, **G**, **I**, **J**, **L**–**T**, **V**, **W**), and two-way repeated-measures ANOVA (**U**). *P < 0.05, **P < 0.01.

To study the effect of METTL16 on learning and memory, we first screened for effective METTL16 knockdown targets in HT22 cells. Puromycin was added after the three lentiviruses Lenti-sh-METTL16-1, Lenti-sh-METTL16-2, and Lenti-sh-METTL16-3 were transfected into HT22 cells. The results of fluorescence microscopy showed that almost all the non-transfected cells were killed (Fig. 2A). Western blotting results showed that both Lenti-sh-METTL16-1 and Lenti-sh-METTL16-3 could knockdown METTL16; however, Lenti-sh-METTL16-3 exhibited the best effect (Fig. 2B, C). Moreover, the qRT–PCR results also showed that the knockdown efficiency of Lenti-sh-METTL16-3 was nearly 65% (Fig. 2D).

Then, the AAV virus with the same target as Lenti-sh-METTL16-3 was prepared and injected into the hippocampi using stereotaxic injections in vivo (Fig. 2E). The results of western blotting and IHC suggested that the expression level of METTL16 was reduced in the AAV-sh-METTL16 group than in the AAV-control group (Fig. 2F–I). The qRT–PCR results also showed that the knockdown efficiency of AAV-sh-METTL16 was nearly 60% (Fig. 2J). Subsequently, the Y-maze, open-field test, object recognition test (ORE), and MWM tests were performed one by one (Fig. 2K). The Y-maze test suggested that METTL16 knockdown in the hippocampi reduced the number of entries and time spent in the new arm (Fig. 2L–M), and prolonged the latency first entrance to the new arm (Fig. 2N); however, it did not affect the total distance (Fig. 2O). The object recognition test (ORT) results suggested that METTL16 knockdown in the hippocampi could reduce the discrimination index (DI) for new objects at 2 and 24 h after excluding the mice’s preference for object location in the training phase (Fig. 2P–R). The MWM test suggested that METTL16 knockdown in the hippocampi prolonged the latency in the training trial phase and reduced the number of platform crossings and the time in target quadrants in the probe trial phase. However, it did not exhibit any effect on the average velocity and latency to identify the platform in the visible platform phase (Fig. 2S–W). In addition, the open-field results showed that METTL16 knockdown did not affect the emotional state of mice (Supplementary Fig. 1). In summary, the above experiments proved that the knockdown of METTL16 in the hippocampi can significantly cause learning and memory deficits without affecting the emotional state of mice.

### Knockdown of METTL16 in the hippocampi disrupts hippocampal dendritic spines

**Fig. 3:**
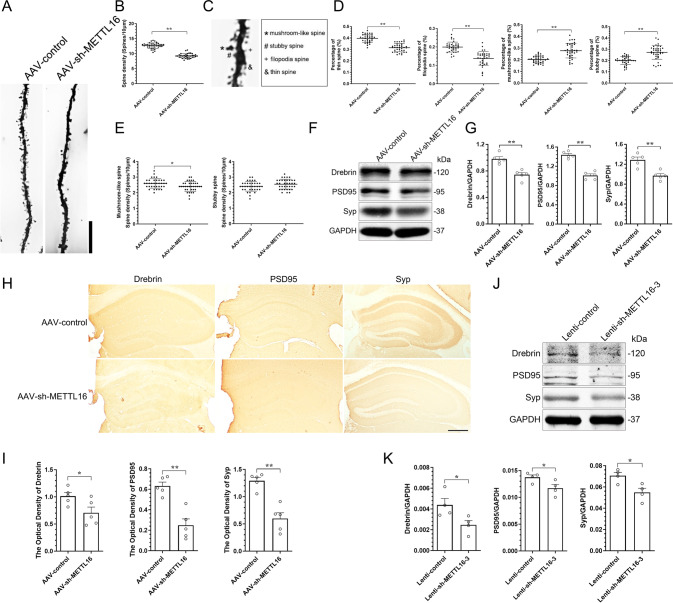
Knockdown of METTL16 in the hippocampi disrupts hippocampal dendritic spines and plasticity-related proteins. **A**, **B** Representative Golgi-Cox impregnated spines (**A**) and quantification of the spine density (**B**) in the hippocampi of the AAV-control and AAV-sh-METTL16 group (*n* = 36 neurons from 6 mice per group); Scale bars = 10 μm. **C**, **D** Representative images of the morphology of mushroom-like, stubby, filopodia, and thin spines (**C**) and quantification of the percentage of tin, filopodia, mushroom-like, and stubby spine (**D**) in the hippocampi of the AAV-control and AAV-sh-METTL16 group (*n* = 36 neurons from 6 mice per group). **E** Quantification of the spine density of mushroom-like and stubby spine in the hippocampi of the AAV-control and AAV-sh-METTL16 group (*n* = 36 neurons from 6 mice per group). **F**, **G** Representative images (**F**) and quantification of Drebrin, PSD95, and Syp (**G**) in the hippocampi of the two groups of mice were tested by western blotting (*n* = 5). **H**, **I** Representative IHC images (**H**) and quantification of Drebrin, PSD95, and Syp (**I**) in the hippocampi of the two groups of mice (*n* = 5); Scale bars = 500 μm. **J**, **K** Representative images (**J**) and quantification of Drebrin, PSD95, and Syp (**K**) in the HT22 cells of the Lenti-control group (*n* = 4) and Lenti-sh-METTL16 group (*n* = 4) tested by western blotting. Data shown as the mean ± SEM. *P-*values were determined by two-tailed *t*-test. *P < 0.05, **P < 0.01.

Synaptic plasticity, that is, the adjustability of synaptic morphology and strength, is considered the basis of learning and memory. Neuronal plasticity is, in part regulated by dendritic spine density and morphology. Thus, Golgi-Cox staining was used to investigate the effects of METTL16 knockdown on the density and morphology of dendritic spines [29] in hippocampal pyramidal neurones. Golgi-Cox staining suggested that METTL16 knockdown in the hippocampi decreased spine density of hippocampal pyramidal dendrites (Fig. 3A, B). Representative images of spine morphology of mushroom-like, stubby, thin, and filopodia were observed using Golgi-Cox staining (Fig. 3C). These four types of dendritic spines were quantified, and the results suggested that METTL16 knockdown in the hippocampi reduced the percentage of spines having thin and filopodia morphology, which promoted an increase in the percentage of mushroom-like and stubby spines (Fig. 3D). Thus, the knockdown of METTL16 might mainly disrupt the generation of thin and filopodia spines. To further clarify the effect of METTL16 knockdown on mushroom and stubby spines, the spine density of mushroom and stubby was examined. The results indicated that the knockdown of METTL16 also disrupted the generation of mushroom spines (Fig. 3E).

Local protein synthesis is critical for dendritic spine formation. Therefore, we examined two post-synaptic plasticity-related proteins (Drebrin and post-synaptic density-95 [PSD95]) and one pre-synaptic plasticity-related protein (synaptophysin [Syp]). Western blotting and IHC showed that METTL16 knockdown in the hippocampi reduced the expression of Drebrin, PSD95, and Syp (Fig. 3F–I). Subsequent experiments at the cellular level showed that METTL16 knockdown in HT22 cells also reduced the expression of Drebrin, PSD95, and Syp (Fig. 3J, K). In summary, the above experiments suggest that the effects of METTL16 on synapses might be extensive in both pre-synaptic and post-synaptic functions.

### Knockdown of METTL16 reduces the overall levels of m^6^A by reducing the expression of MAT2A

**Fig. 4:**
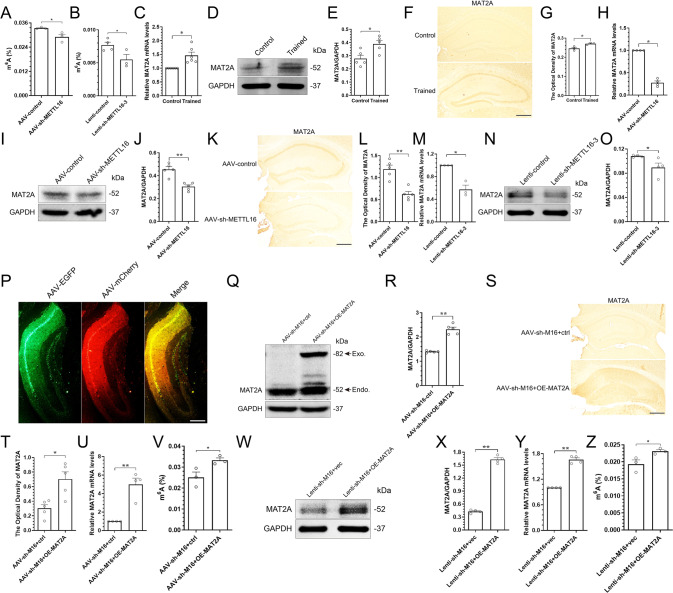
Knockdown of METTL16 reduces the overall levels of m^6^A by reducing the expression of MAT2A. **A** Overall levels of m^6^A in the hippocampi of the AAV-control (*n* = 3) and AAV-sh-METTL16 group (*n* = 3) were tested by m^6^A colorimetry. **B** Overall levels of m^6^A in the HT22 cells of the Lenti-control group (*n* = 4) and Lenti-sh-METTL16-3 group (*n* = 3) were tested by m^6^A colorimetry. **C** Expression level of MAT2A mRNA was tested by qRT–PCR in the hippocampi of MWM-trained (*n* = 6) and untrained mice (*n* = 6). **D**, **E** Representative images (**D**) and quantification of MAT2A (**E**) in the hippocampi of MWM-trained (*n* = 5) and untrained mice (*n* = 5) tested by western blotting. **F**, **G** Representative IHC images (**F**) and quantification of MAT2A (**G**) in the hippocampi of MWM-trained (*n* = 3) and untrained mice (*n* = 3); Scale bars = 500 μm. **H** Expression level of MAT2A mRNA was tested by qRT–PCR in the hippocampi of the AAV-control group (*n* = 3) and AAV-sh-METTL16 group mice (*n* = 3). **I**, **J** Representative images (**I**) and quantification of MAT2A (**J**) in the hippocampi of the AAV-control group (*n* = 5) and AAV-sh-METTL16 group mice (*n* = 5) tested by western blotting. **K**, **L** Representative IHC images (**K**) and quantification of MAT2A (**L**) in the hippocampi of the AAV-control group (*n* = 5) and AAV-sh-METTL16 group mice (*n* = 5); Scale bars = 500 μm. **M** Expression level of MAT2A mRNA was tested by qRT–PCR in the HT22 cells of the Lenti-control group (*n* = 3) and Lenti-sh-METTL16-3 group (*n* = 3). **N**, **O** Representative images (**N**) and quantification of MAT2A (**O**) in the HT22 cells of the Lenti-control group (*n* = 4) and Lenti-sh-METTL16-3 group (*n* = 4) tested by western blotting. **P** Representative image of stereotactic injections of AAV-sh-M16+OE-MAT2A in the hippocampi; Scale bars = 500 μm. **Q**, **R** Representative images (**Q**) and quantification of MAT2A (**R**) in the hippocampi of the AAV-sh-M16+ctrl group (*n* = 5) and AAV-sh-M16+OE-MAT2A group mice (*n* = 5) tested by western blotting. **S**, **T** Representative IHC images (**S**) and quantification of MAT2A (**T**) in the hippocampi of the AAV-sh-M16+ctrl group (*n* = 5) and AAV-sh-M16+OE-MAT2A group mice (*n* = 5); Scale bars = 500 μm. **U** Overexpression efficiency of MAT2A in the hippocampi was detected by qRT–PCR (*n* = 4). **V** Overall levels of m^6^A in the hippocampi of the AAV-sh-M16+ctrl group (*n* = 3) and AAV-sh-M16+OE-MAT2A group mice (*n* = 3) tested by m^6^A colorimetry. **W**, **X** Representative images (**W**) and quantification of MAT2A (**X**) in the HT22 cells of the Lenti-sh-M16+vec group (*n* = 4) and Lenti-sh-M16+OE-MAT2A group (*n* = 4) tested by western blotting. **Y** Overexpression efficiency of MAT2A in the HT22 cells was detected by qRT–PCR. **Z** Overall levels of m^6^A in the HT22 cells of the Lenti-sh-M16+vec group (*n* = 4) and Lenti-sh-M16+OE-MAT2A group (*n* = 3) were tested by m^6^A colorimetry. Data shown as the mean ± SEM. *P*-values were determined by two-tailed *t*-test. *P < 0.05, **P < 0.01.

After clarifying the effects of METTL16 knockdown on learning, memory and hippocampal dendritic spines, the m^6^A colourimetric quantification at animal and cellular levels revealed that these memory decays might be associated with decreased overall levels of m^6^A (Fig. 4A, B), which is consistent with the study by Pendleton et al. [23]. It has been reported that METTL16 had a limited number of validated direct targets, including MAT2A mRNA, U6 snRNA and long non-coding RNA (lncRNA) MALAT [30, 31], which distinguishes it from the METTL3/METTL14 complex, who modifies most m^6^A sites on RNA [24, 32]. Therefore, the knockdown of METTL16 is unlikely to reduce the overall levels of m^6^A by directly affecting the number of m^6^A modifications in its target RNAs. Instead, this may be due to the specific properties of METTL16’s target genes to regulate the overall levels of m^6^A. In this context, one of METTL16’s targets, MAT2A, has caught our attention. MAT2A is a key enzyme for methyl donor synthesis, which has important implications for the maintenance of m^6^A methylation homoeostasis [20–24]. Decreased m^6^A levels are associated with learning and memory deficits [33]. Collectively, the learning and memory deficits caused by METTL16 knockdown may be related to the decreased expression of MAT2A.

Therefore, the expression levels of MAT2A were detected using both animal and cellular experiments. The results of animal experiments showed that the expression of MAT2A mRNA and protein in the hippocampi of MWM-trained mice increased with the upregulation of METTL16 (Fig. 4C–G), and METTL16 knockdown in the hippocampi reduced the expression of MAT2A mRNA and protein (Fig. 4H–L). The results of cellular experiments also showed that METTL16 knockdown reduced the expression of MAT2A mRNA and protein in HT22 cells (Fig. 4M–O). Thus, METTL16 knockdown might inhibit the expression of MAT2A protein by reducing the amount of MAT2A mRNA, which might contribute to the reduction of the overall levels of m^6^A.

### Overexpression of MAT2A in the hippocampi increases the overall levels of m^6^A reduced by METTL16 knockdown

To further demonstrate the regulation of MAT2A on the overall level of m^6^A, we overexpressed MAT2A in the hippocampi of METTL16 knockdown (Fig. 4P). Western blotting and IHC showed that the expression level of MAT2A increased significantly in the AAV-sh-M16 + OE-MAT2A group compared to that in the AAV-sh-M16 + ctrl group (Fig. 4Q–T). Moreover, qRT–PCR results showed that the overexpression efficiency of MAT2A was nearly five times that of the control group (Fig. 4U). Subsequently, RNA m^6^A quantification experiments showed that MAT2A overexpression in the hippocampi of METTL16 knockdown mice increased the overall levels of m^6^A (Fig. 4V). The results of cellular experiments also showed that MAT2A overexpression in HT22 cells with METTL16 knockdown increased the overall levels of m^6^A (Fig. 4W–Z).

### Overexpression of MAT2A in the hippocampi improves the hippocampal dendritic spines disrupted by METTL16 knockdown

**Fig. 5:**
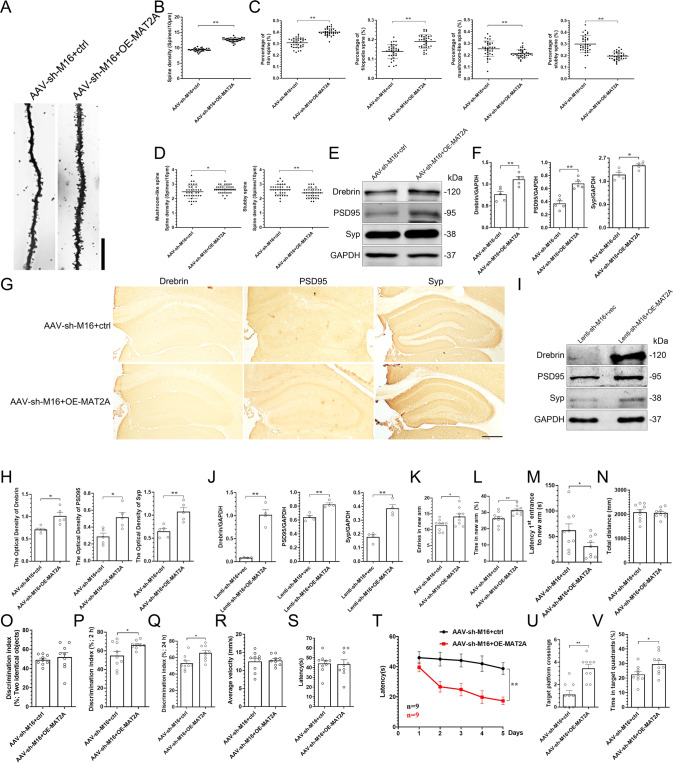
Overexpression of MAT2A in hippocampi improves the hippocampal learning and memory disrupted by METTL16 knockdown. **A** Representative Golgi-Cox impregnated spines in the hippocampi of the AAV-sh-M16+ctrl and AAV-sh-M16+OE-MAT2A group; Scale bars = 10 μm. **B**, **C** Quantification of the spine density (**B**) and the percentage of tin spine, filopodia spine, mushroom-like spine, and stubby spine (**C**) in the hippocampi of the two groups of mice (*n* = 36 neurons from 6 mice per group). **D** Quantification of the spine density of mushroom-like and stubby spine in the hippocampi of the two groups of mice (*n* = 36 neurons from 6 mice per group). **E**, **F** Representative images (**E**) and quantification of Drebrin, PSD95, and Syp (**F**) in the hippocampi of the two groups of mice were tested by western blotting (*n* = 5). **G**, **H** Representative IHC images (**G**) and quantification of Drebrin, PSD95, and Syp (**H**) in the hippocampi of the two groups of mice (*n* = 5); Scale bars = 500 μm. **I**, **J** Representative images (**I**) and quantification of Drebrin, PSD95, and Syp (**J**) in the HT22 cells of the Lenti-sh-M16+vec group (*n* = 4) and Lenti-sh-M16+OE-MAT2A group (*n* = 4) tested by western blotting. **K**–**N** Entries in new arm (**K**), time in new arm (**L**), latency 1st entrance to new arm (**M**), and total distance (**N**) of mice in the AAV-sh-M16+ctrl (*n* = 9) and AAV-sh-M16+OE-MAT2A group (*n* = 9) were tested in Y-maze test. **O**–**Q** Discrimination index (DI) for new objects of mice in the AAV-sh-M16+ctrl (*n* = 9) and AAV-sh-M16+OE-MAT2A group (*n* = 9) was tested in 2 h (**P**) and 24 h (**Q**) after excluding the mice’s preference for object location (**O**) in the ORT experiment. **R**, **S** Average velocity (**R**) and latency to find the platform (**S**) of mice in the AAV-sh-M16+ctrl (*n* = 9) and AAV-sh-M16+OE-MAT2A group (*n* = 9) were explored in the visible platform phase of MWM. **T** Latency to find the platform of mice in the AAV-sh-M16+ctrl (*n* = 9) and AAV-sh-M16+OE-MAT2A group (*n* = 9) were explored in the training trials phase of MWM. **U**, **V** Number of platform crossings (**U**) and time in target quadrants (**V**) of mice in the AAV-sh-M16+ctrl (*n* = 9) and AAV-sh-M16+OE-MAT2A group (*n* = 9) were explored in the probe trial phase of MWM. Data shown as the mean ± SEM. *P-*values were determined by two-tailed *t*-test (**B**–**D**, **F**, **H**, **J**–**S**, **U**, **V**), and two-way repeated-measures ANOVA (**T**). *P < 0.05, **P < 0.01.

After elucidating the function of MAT2A in the overall m^6^A levels deficits caused by METTL16 knockdown, we investigated whether MAT2A mediates the function of METTL16 on the plasticity of dendritic spine. Golgi-Cox staining suggested that MAT2A overexpression in the hippocampi of METTL16 knockdown increased spine density (Fig. 5A, B) and the percentage of spines exhibiting thin and filopodia morphology in hippocampal pyramidal dendrites, which reduced the percentage of mushroom-like and stubby spines (Fig. 5C). Thus, the overexpression of MAT2A in the hippocampi of METTL16 knockdown might mainly promote the generation of thin and filopodia spines. To further clarify the effect of MAT2A overexpression on mushroom and stubby spines, the spine density of mushroom and stubby was examined. The results indicated that the overexpression of MAT2A in the hippocampi of METTL16 knockdown also promoted the generation of mushroom spines (Fig. 5D). Western blotting and IHC results showed that MAT2A overexpression in the hippocampi of METTL16 knockdown mice increased the expression levels of Drebrin, PSD95, and Syp (Fig. 5E–H). Subsequent experiments at the cellular level also showed that MAT2A overexpression in HT22 cells with METTL16 knockdown increased the expression levels of Drebrin, PSD95, and Syp (Fig. 5I, J). In summary, the above experiments suggest that MAT2A mediates the function of METTL16 in dendritic spine formation by promoting the expression of both pre-synaptic and post-synaptic plasticity-related proteins.

### Overexpression of MAT2A in the hippocampi improves the learning and memory deficit caused by METTL16 knockdown

The Y-maze test suggested that MAT2A overexpression in the hippocampi of METTL16 knockdown mice increased the number of entries and time in the new arm (Fig. 5K, L), and shortened the latency first entrance to the new arm (Fig. 5M); however, it did not affect the total distance (Fig. 5N). The ORT results suggested that MAT2A overexpression in the hippocampi of METTL16 knockdown mice could increase the DI for new objects at 2 and 24 h after excluding the mice’s preference for object location in the training phase (Fig. 5O–Q). The MWM test suggested that MAT2A overexpression in the hippocampi of METTL16 knockdown mice shortened the latency in the training trial phase and increased the number of platform crossings and the time in target quadrants in the probe trial phase. However, it did not exhibit any effect on the average velocity and latency to identify the platform in the visible platform phase (Fig. 5R–V). In addition, the open-field test showed that MAT2A did not affect the emotional state of mice (Supplementary Fig. 2). In summary, the above experiments proved that the overexpression of MAT2A in the hippocampi of METTL16 knockdown mice significantly alleviated learning and memory deficits caused by METTL16 knockdown without affecting the emotional state of mice.

### METTL16 promotes the stability of MAT2A mRNA

**Fig. 6:**
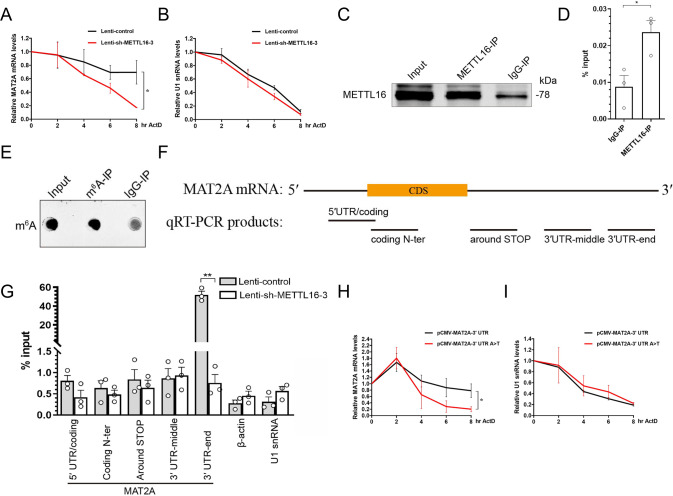
METTL16 and the METTL16 methylation site on the 3′UTR-end of MAT2A mRNA is required for the stability of MAT2A mRNA. **A**, **B** HT22 cells were treated with 5 μg/ml ActD for up to 8 h with or without prior knockdown of METTL16. The expression levels of MAT2A mRNA (**A**) and U1 snRNA (**B**) were measured on the 0, 2, 4, 6, and 8 h after the ActD addition (*n* = 3). **C**, **D** RIP-qRT–PCR was employed to investigate the direct interaction of METTL16 protein and MAT2A mRNA in the HT22 cells. Immunoblot analysis of METTL16 was performed as a control (**C**). MAT2A mRNA was assessed by qRT–PCR in endogenous METTL16, or IgG (negative control) immunoprecipitates from HT22 cells and are shown as percentages of input RNA (*n* = 3) (**D**). **E**–**G** Anti-m^6^A RIP-qRT–PCR using total RNA from HT22 cells with or without METTL16 knockdown. Immunoblot analysis of m^6^A was performed as a control (**E**). Cartoon render of the PCR products in MAT2A mRNA (**F**). The indicated transcripts of MAT2A mRNA (**F**), β-actin, and U1 snRNA were quantified by qRT–PCR and are shown as percentages of input RNA (**G**) (*n* = 3). **H**, **I** HT22 cells were treated with 5 μg/ml ActD for up to 8 h with prior overexpression of MAT2A mRNA-3′UTR-wild or MAT2A mRNA-3′UTR-mut. The expression levels of MAT2A mRNA (**H**) and U1 snRNA (**I**) were measured on the 0, 2, 4, 6, and 8 h after the ActD addition (*n* = 3). Data shown as the mean ± SEM. *P*-values were determined by two-tailed *t*-test (**D**, **G**) and two-way repeated-measures ANOVA (**A**, **B**, **H**, **I**). *P < 0.05, **P < 0.01.

For the mechanism of METTL16 regulating MAT2A expression, Shima et al. [21] found that METTL16 is required for the stability of MAT2A mRNA. Therefore, we investigated if our study could corroborate this finding. The transcription inhibitor ActD was used to test the stability of MAT2A mRNA. Knockdown of METTL16 before ActD addition significantly accelerated MAT2A mRNA degradation, that is, 8 h after ActD addition, ~70% of the initial amount reduced to ~20% (Fig. 6A). In contrast, we observed no effect of the METTL16 knockdown on U1 snRNA degradation (Fig. 6B). The results of the experiments described above suggest that MAT2A mRNA was stabilised if METTL16 was sufficient, which partially contributed to an increase in the abundance of mRNA.

### The METTL16 methylation site on the 3′-UTR-end of MAT2A mRNA is required for the stability of MAT2A mRNA

To study the mechanism by which METTL16 enhances the stability of MAT2A mRNA, we investigated the direct interaction between the METTL16 protein and MAT2A mRNA in HT22 cells. After confirming the practicality of the anti-METTL16 antibody (Fig. 6C), MAT2A mRNA adsorbed by METTL16 was tested using qRT–PCR. Enrichment of MAT2A mRNA was observed in the samples immunoprecipitated with the anti-METTL16 antibody than with the IgG-IP control antibody (Fig. 6D), suggesting that METTL16 interacted with MAT2A mRNA in HT22 cells. After confirming the practicality of the anti-m^6^A antibody (Fig. 6E), we investigated the METTL16 methylation site of MAT2A mRNA. RNA immunoprecipitation (IP) was performed using an anti-m^6^A antibody (anti-m^6^A RIP). We found that the 3′UTR-end of MAT2A mRNA precipitated more efficiently than the other regions (Fig. 6F, G), indicating the presence of more m^6^A sites and/or a stoichiometrically higher ratio of m^6^A modifications in this region. Importantly, m^6^A levels in this region were greatly reduced in METTL16 knockdown cells (Fig. 6G), whereas the levels of m^6^A were not altered by METTL16 knockdown in other regions of MAT2A (the regions of 3′-UTR/coding, coding N-ter, around STOP codons, and 3′-UTR-middle in MAT2A mRNA) or other RNAs not catalysed by METTL16 (β-actin and U1 snRNA) (Fig. 6G). In summary, the above experiments suggested that the METTL16 methylation site on the end of MAT2A mRNA-3′-UTR was present.

We further examined whether this METTL16 methylation site on the 3′UTR-end of MAT2A mRNA is required for the stability of MAT2A mRNA. The ‘TACAGAGAA’ nonamer is considered a conserved region recognised by METTL16, and the ‘A’ base at position 4 is its methylation site [23]. Therefore, the mutant-MAT2A-3′UTR (A > T) and wild-MAT2A-3′UTR expression plasmids were separately transfected into HT22 cells, and the stability of MAT2A mRNA was assessed. The results showed that the mutant significantly accelerated MAT2A mRNA degradation, that is, 8 h after ActD addition, ~75% of the initial amount reduced to ~20% (Fig. 6H). In contrast, we observed no effect of the mutant on U1 snRNA degradation (Fig. 6I). These results suggested that MAT2A mRNA was stabilised when the 3′UTR-end was occupied by m^6^A, which partially contributed to an increase in the abundance of mRNA.

## Discussion

Short- and long-term memories are subject to multiple layers of control, from post-translational modification at synapse to the production of RNA in nucleus [5, 6, 34]. The stability and translation of RNA transcripts including mRNA, microRNA (miRNA), and lncRNA have been observed to depend on RNA m^6^A methylation [35], which is strongly biased towards neuronal RNA [36, 37]. Thus, m^6^A modification has been associated with learning and memory [9, 38]. In this study, we focused on the roles and regulatory mechanisms of the m^6^A methyltransferase METTL16 in learning and memory as METTL16 and m^6^A levels were significantly upregulated in the hippocampi of MWM-trained mice.

Although the roles of m^6^A methyltransferases in learning and memory have been reported in many studies, ours is the first to demonstrate the function of METTL16 in learning and memory. METTL3, an extensively studied methyltransferase, has been implicated in the formation of hippocampal-dependent memory directly [39]. Particularly, overexpression of METTL3 in the dorsal hippocampi was shown to have a strategic role in consolidating long-term memory [40], whereas knockout of METTL3 in the forebrain was shown to have a negative effect during memory consolidation [33, 40]. METTL14-mediated RNA m^6^A modification is also essential for epitranscriptomic regulation of memory formation. Conditional METTL14 knockdown reduces striatal m^6^A levels, increases neuronal sensitivity to dopaminergic drugs, and severely impairs striatal-mediated learning-related behaviour [41]. More importantly, in this study, the learning and memory behaviours and hippocampal dendritic spines of mice administered with stereotaxic injection were found to be destroyed after METTL16 knockdown in the hippocampi. Synaptic plasticity-related proteins, including Drebrin, PSD95, and Syp, were also found to be reduced after METTL16 knockdown in the hippocampi and HT22 cells. Therefore, tantamount to METTL3 and METTL14, METTL16 may be another methyltransferase that improves learning and memory deficits.

METTL16 is unable to form a complex with other m^6^A methyltransferases, unlike METTL3 and METTL14, and has a limited number of m^6^A modification targets, including MAT2A mRNA, U6 snRNA, and lncRNA MALAT [30, 31]. Interestingly, knockdown of METTL16 was found to reduce the overall levels of m^6^A [23], which may be attributed to METTL16-targeting MAT2A, a key enzyme for methyl donor synthesis [20–24]. The present study confirmed that METTL16 knockdown in the hippocampi and HT22 cells downregulated m^6^A levels and the expression of MAT2A, while MAT2A overexpression in the hippocampi and HT22 cells of METTL16 knockdown upregulated m^6^A levels.

Decreased m^6^A levels are associated with learning and memory deficits [33]. On the contrary, increased m^6^A levels in the hippocampi of MWM-trained mice or MAT2A overexpression mice were found improve learning and memory in our study. Specific manifestation is as follows: The disorders of learning and memory behaviour and hippocampal dendritic spines were improved after MAT2A overexpression in the hippocampi of METTL16 knockdown mice. The synaptic plasticity-related proteins, including Drebrin, PSD95, and Syp, were also found to be upregulated after MAT2A overexpression in the hippocampi of METTL16 knockdown mice or HT22 cells.

Our findings on the mechanism of METTL16-targeting MAT2A indicated that the stability of MAT2A mRNA decreased after METTL16 knockdown. This is consistent with the finding of Shima et al. [21] that METTL16 is necessary to maintain the stability of MAT2A mRNA. To further demonstrate the mechanism by which METTL16 regulates the stability of MAT2A mRNA, we identified METTL16 methylation sites in MAT2A mRNA. Interestingly, although previous studies have revealed that m^6^A is preferentially present in close vicinity to the termination codon of mRNA [36, 42, 43], our study did not discern such a preference of m^6^A around the termination codon of MAT2A mRNA as compared to that in other regions, nor was it affected by METTL16 knockdown. Moreover, there was only one ‘TACAGAGAA’ nonamer recognised by METTL16 on the 3ʹUTR-end of MAT2A mRNA, which did not corroborate the finding of Pendleton et al. [23] that METTL16 could promote m^6^A methylation at six ‘TACAGAGAA’ nonamer sites on the 3ʹUTR of MAT2A mRNA. This difference suggests the presence of a unique type of regulation in hippocampus. The absence of m^6^A methylation on the other five METTL16 recognition sites remains elusive and should be investigated in future studies. Anyway, our results suggest that the 3ʹUTR-end of MAT2A mRNA is an internal methylation-sensitive site, which highly prone to m^6^A methylation or off-target methylation when METTL16 sufficiency or not. Subsequent site mutation experiments also proved this point of view, as the single base mutation of this methylation site reduced the stability of MAT2A mRNA. It is well known that MAT2A, as a key enzyme for the generation of methylation donors, is essential for the maintenance of methylation homoeostasis [20–24, 44]. Therefore, we speculate that the high sensitivity of this methylation site in the 3ʹUTR-end of MAT2A mRNA may be important for the maintenance of intracellular methylation homoeostasis.

## Conclusion

In this study, METTL16 was upregulated in the hippocampi of MWM-trained mice. Artificial knockdown of METTL16 in the hippocampi has been found to result in synaptic disorders and memory impairments by inhibiting the stability of MAT2A mRNA. The stability of MAT2A mRNA is associated with the m^6^A methylation site of METTL16 on the 3ʹ-UTR-end of MAT2A mRNA. Overexpression of MAT2A rescued disrupted dendritic spine morphology and memory impairment by METTL16 knockdown. In summary, our study demonstrated that METTL16 in hippocampus can increase the expression of MAT2A by promoting m^6^A methylation of the 3ʹUTR-end of MAT2A mRNA and increasing the stability of MAT2A mRNA, thereby elevating m^6^A levels and subsequently accelerating the formation of synaptic plasticity and enhancing learning and memory.

## Materials and methods

### Animals and cell culture

Adult male C57BL/6J mice (6–8 weeks old, 20–24 g) were purchased from Hua Fukang Biological Technologies (Beijing, China). Four–five mice per cage were fed ad libitum and housed in a controlled environment of temperature. The light/dark cycle was 12 h light/12 h dark. Immortalised mouse hippocampal HT22 cells were purchased from Wuhan Pu-nuo-sai Life Technology Co. Ltd. (Wuhan, China), and cultured as previously described [45]. When confluence was close to 70–80%, the adherent cells in 10 cm culture dishes were digested with 0.25% pancreatin and subcultured. Specifically, the cells were seeded at 50,000 cells/cm^2^ in six-well plates and cultured for another day for transfection assays.

All animal experiments in this study were complied with the guidelines of the Animal Welfare Act of the National Institutes of Health Guide for the Care and Use of Laboratory Animals (NIH Publication No. 85–23, revised 1996), and were approved by the Ethics Committee of Hebei Medical University (IACUC-Hebmu-Glp-2,016,017). All the investigators were blinded and the study was not preregistered.

### MWM training

Three-month-old C57BL/6J mice were used for MWM training. The mice were trained for 5 consecutive days in a black circular tank (diameter: 1.2 m and height: 0.6 m) to find a hidden platform (10 cm in diameter), with four trials (>30 min interval) per day from 08:00 a.m. to 04:00 p.m. Each trial began with mice from one of the four quadrants with their backs to the wall of the pool and ended when they climbed onto the escape platform. A trial terminated when the mice climbed onto the platform and remained on it for at least 5 s. If the mice did not climb onto the platform in 60 s, they would guide to the platform. After 5 days of training, the hippocampal tissues of the trained mice were collected for proteomic data analysis (*n* = 3), western blotting (*n* = 5), IHC staining (*n* = 3), and RNA m^6^A quantification (*n* = 3).

### Proteomic data analysis

Quantitative proteomic data analysis was performed using tandem mass tag (TMT) technology in Shanghai Applied Protein Technology (APT, Shanghai, China). Briefly, each 100 μg peptide mixture was digested with trypsin (Promega, Madison, WI, USA) and labelled using TMT reagent according to the manufacturer’s instructions (Thermo Fisher Scientific, Waltham, MA, USA). Labelled peptides were further purified by strong cation-exchange chromatography using an AKTA Purifier system (GE Healthcare Life Sciences, Uppsala, Sweden). Liquid chromatography with tandem mass spectrometry analysis was used on a Q Exactive mass spectrometer (Thermo Fisher Scientific) coupled to an Easy nLC (Thermo Fisher Scientific) for 60 min. The mass spectrometer was functioned in the positive-ion mode. MS data were obtained using a data-dependent top10 method dynamically choosing the most abundant precursor ions from the survey scan (300–1800 *m/z*) for higher-energy C-trap dissociation (HCD) fragmentation. The automatic gain control target and maximum injection time were set as 3E6 and 10 ms, separately. The dynamic exclusion duration was set to 40.0 s. Survey scans were obtained at a resolution of 70,000 at *m/z* 200, the HCD spectra were acquired at a resolution of 17,500 at *m/z* 200, and the isolation width was 2 *m/z*. The normalised collision energy was 30 eV, and the underfill ratio was defined as 0.1%. The instrument was operated in the peptide recognition mode. The MS raw data for each sample were searched using the MASCOT engine (Matrix Science, London, UK; v2.2) embedded in Proteome Discoverer 1.4 software for identification and quantification analysis. The related parameters and instructions are listed in Supplementary Table 1.

### Western blotting

To prepare the protein samples, the isolated hippocampal tissue or HT22 cell samples were homogenised and lysed in RIPA buffer (#R0020, Solarbio, China) containing protease inhibitor (PMSF, #PO100, Solarbio, China). The supernatant was collected after the homogenate was centrifuged at 12,000 rpm for 20 min at 4 °C. The concentration of the proteins was measured using a NanoDrop Lite spectrophotometer (Thermo Fisher Scientific). The protein samples above were mixed with 5× loading buffer (#BL502A; Biosharp, China), boiled, and 20 μg of protein was separated on a 10% sodium dodecyl sulfate (SDS) polyacrylamide gel and transferred to polyvinylidene fluoride (PVDF) membranes by electroblotting for 2.5 h at 200 mA. 5% milk was used for blocking. The membranes were then incubated with the following primary antibodies: rabbit anti-METTL16 (1:1,000, #17676, Cell Signaling Technology, USA), rabbit anti-METTL16 (1:1,000, #TA504710, ORIGENE, USA), rabbit anti-MAT2A (1:1,000, #NBP1-92100, Novus, USA), rabbit anti-Drebrin (1:2,000, #10260-1-AP, Proteintech, China), rabbit anti-PSD95 (1:2,000, #ab18258, Abcam, USA), rabbit anti-Syp (1:2,000, #CY5273, Abways, China), rabbit anti-GAPDH (1:10,000, #ab9485, Abcam, USA), or mouse anti-GAPDH (1:50,000, #60004–1-Ig, Proteintech, China) at 4 °C overnight. The membranes were then washed thrice in Tris-buffered saline with 0.1% Tween-20 (TBST) for 5 min each and blotted with the secondary antibody anti-mouse IgG (H&L) (GOAT) antibody IRDye® 700 conjugated (1:10,000, #610–130-121, ROCKLAND, USA) or anti-rabbit IgG (H&L) (GOAT) antibody Dylight™ 800 conjugated (1:10,000, #611–145-002, ROCKLAND, USA) for 2 h at room temperature (~25 °C). After washing again in TBST, the reactive bands were visualised using an Odyssey IR fluorescence scanning imaging system (LICOR, USA) and analysed using ImageJ 1.51j8 for grey values. The formula performed to calculate relative target protein expression was as follows: Relative expression of target protein = Grey value of target protein band/grey value of GAPDH.

### Immunohistochemical staining (IHC) staining

Mice were anaesthetised with 1% pentobarbital sodium by intraperitoneal injection (0.1 mL/20 g) and transcardially perfused with 40 mL chilled 0.9% saline solution, then perfused with 40 mL chilled 4% paraformaldehyde. After perfusion, the brains were extracted immediately and fixed in 4% paraformaldehyde for 24 h prior to being embedded in paraffin. IHC was performed using the streptavidin–peroxidase (SP) method (Biotin–Streptavidin Immunohistochemistry Kit; #SP-9001; ZSGB-Bio, China). Sections (10 µm thick) were prepared from paraffin-embedded tissue blocks, mounted on microscope slides, placed in a 65 °C oven to bake slices, dewaxed with xylene, and rehydrated with graded ethanol. For antigen retrieval, the sections were immersed in citric acid buffer, placed in microwave for 7 min, and cooled down to room temperature (~25 °C). Endogenous peroxidase activity was blocked using 0.3% H_2_O_2_ from the kit (liquid A) for 30 min, followed by washing thrice in phosphate-buffered saline (PBS). The sections were then blocked with the blocking serum from the kit (liquid B) at room temperature (~25 °C) for 30 min and incubated with the following primary antibodies: rabbit anti-METTL16 (1:100, #ab221036, Abcam, USA), rabbit anti-MAT2A (1:200, #NBP1-92100, Novus, USA), rabbit anti-Drebrin (1:200, #10260-1-AP, Proteintech, China), rabbit anti-PSD95 (1:200, #ab18258, Abcam, UK), or rabbit anti-Syp (1:200, #CY5273, Abways, China) at 4 °C overnight. Subsequently, the sections were incubated with the biotinylated secondary antibody (room temperature, 30 min, liquid C), horseradish peroxidase (room temperature, 60 min, liquid D), and 3,3-diaminobenzidine chromogen (room temperature, 3 min, #ZLI-9018, ZSGB-Bio, China). Following dehydration with gradient ethanol, the sections were made transparent with xylene and sealed with neutral balsam. Observations were made using an Olympus light microscope (BX43; Olympus, Japan) and images were analysed with ImageJ 1.51j8 to obtain the mean optical density.

### RNA m^6^A quantification

Total RNA from the isolated hippocampal tissues or HT22 cell samples was extracted using the Total RNApure Kit (#ZP404-1, ZOMANBIO, China) according to the manufacturer’s protocols and quantified using a NanoDrop Lite spectrophotometer (Thermo Fisher Scientific). Further, the m^6^A level was identified using the EpiQuik m^6^A RNA Methylation Colourimetric kit (#P-9005, AmyJet Scientific, USA). Briefly, 300 ng RNA sample with 80 μL RNA high binding solution (BS) was added dropwise to strip wells and incubated for 90 min at 37 °C. After washing thrice with wash buffer (WB), the capture antibody (CA), detection antibody (DA), and enhancer solution (ES) were added sequentially and incubated separately for 1 h at room temperature (approximately 25 °C). After washing five times in WB, the wells were incubated with 100 μL colour-developing solution (DS) and protected from light for ~6 min at which time the solution turned blue in the m^6^A positive wells, then 100 μL stop solution (SS) was added to arrest the reaction. Finally, the absorbance of the stable yellow colour was determined at 450 nm using SoftMax Pro 7 software (SMP7, Molecular Devices, USA). The percentage of m^6^A in the samples was calculated using the following formula:

$${\mathrm{m}}^6{\mathrm{A}}{{{\% }}} = \frac{{\left( {{\mathrm{Sample}}\;{\mathrm{OD}} - {\mathrm{NC}}\;{\mathrm{OD}}} \right) \div 300}}{{{\mathrm{PC}}\;{\mathrm{OD}} - {\mathrm{NC}}\;{\mathrm{OD}}}} \times 100{{{\% }}}$$

where NC is the negative control and PC is the positive control).

### Real-time RT–PCR (qRT–PCR)

In order to evaluate RNA expression levels of METTL16, MAT2A, and U1 snRNA, total RNA from dissected hippocampal tissues and HT22 cells was extracted using the Total RNApure Kit (#ZP404-1, ZOMANBIO, China) and quantified using a NanoDrop Lite spectrophotometer (Thermo Fisher Scientific) based on the protocols provided by the manufacturer. First-strand cDNA was synthesised from 1 μg total RNA using GoScript^TM^ Reverse Transcription System (#A5001; Promega Corporation, USA) at a total volume of 20 μL under the following conditions: 42 °C for 15 min, followed by a termination step at 70 °C for 15 min. The PCR reaction was performed using the GoTaq qPCR Master Mix (#A600A, Promega Corporation, USA) with β-actin for normalisation. Samples were incubated at 95 °C for 2 min, followed by 40 cycles, consisting of a 15 s denaturation step at 95 °C, and a 1 min annealing step at 60 °C. All PCR reactions including controls were run in triplicate reactions. The cycle threshold (Ct value) was obtained using the QuantStudio™ 6 Flex Real-Time PCR System (#4484642, Applied Biosystems, USA). Relative mRNA expression was calculated using the comparative method Ct (Method 2^(−ΔΔCt)^) [ΔCt = Ct (target gene) − ΔCt ( β-actin); ΔΔCt = ΔCt (experimental group) − ΔCt (control group)]. *P*-values for ΔCt was calculated. Table 1 shows the sequences of the primers.

**Table 1:** The primers used in qRT–PCR experiments.

Name		Primer
METTL16	Forward:	5′ GACAAACCACCTGACTTCGCA 3′
	Reverse:	5′ TCTGACTGCTTCGGGGTCTT 3′
MAT2A	Forward:	5′ GCTTCCACGAGGCGTTCAT 3′
	Reverse:	5′ AGCATCACTGATTTGGTCACAA 3′
MAT2A 5’UTR/coding	Forward:	5′ GAAGCGATCCTCCCTCTGTG 3′
	Reverse:	5′ TCAATGAACGCCTCGTGGAA 3′
MAT2A conding N-ter	Forward:	5′ TCCACGAGGCGTTCATTGAG 3′
	Reverse:	5′ TGCATCAAGGACAGCATCACT 3′
MAT2A around STOP	Forward:	5′ CCACTTTGGTAGGGACAGCTT 3′
	Reverse:	5′ GGCCCTTTCCCTCAGAGCTT 3′
MAT2A 3’UTR-middle	Forward:	5′ GTCACAGGGCAGTACCTGAG 3′
	Reverse:	5′ CCCTGGGAGGAGCTATTGTG 3′
MAT2A 3’UTR-end	Forward:	5′ GGGTTAGACCTACAGGGGGT 3′
	Reverse:	5′ TTGCTTAGGGCAAGCAGTCA 3′
U1 snRNA	Forward:	5′ GGCTTATCCATTGCACTCCG 3′
	Reverse:	5′ AGTCCCCCACTACCACAAAT 3′
β-actin	Forward:	5′ TCATCACTATTGGCAACGAGCGGT 3′
	Reverse:	5′ GTGTTGGCATAGAGGTCTTTACG 3′

### Knockdown of METTL16 in HT22 cells

Three METTL16-targeting short hairpin RNAs (shRNAs) were packaged between AgeI and EcoRI of the hU6-MCS-CBh-gcGFP-IRES-puromycin lentiviruses (Supplementary Fig. 3A, Genechem Inc.) designated as Lenti-sh-METTL16-1, Lenti-sh-METTL16-2, and Lenti-sh-METTL16-3. A no-load shRNA lentivirus was used as a control. These recombinant lentiviruses and corresponding control lentiviruses (multiplicity of infection = 80) were transfected into 30% confluent HT22 cells. Further, 48 h post-transfection, non-transfected cells were killed with 4.5 μg/mL puromycin (#HY-B1743A/CS-6857, MCE, China). The efficiency of cell transfection was observed using a fluorescence microscope (Olympus FV1200, Japan). After all the non-transfected cells were killed, the cells were cultured in a normal medium without puromycin. The knockdown efficiency was determined 72 h post-transfection using western blotting and qRT–PCR as mentioned above. Table 2 shows the target sequences of the shRNAs.

**Table 2:** The target sequence of short hairpin RNAs (shRNAs).

Name	Target sequence
Sh-METTL16-1	5′ ACTTACGTACGTAACCAAA 3′
Sh-METTL16-2	5′ AACCTTAAATGGCTGGTAT 3′
Sh-METTL16-3	5′ CTATTTCAGAACCAGAGTT 3′

### Overexpression of MAT2A in HT22 cells

To overexpress MAT2A, the coding sequence (CDS) of MAT2A mRNA was inserted between BamHI and HindIII of the cytomegalovirus (CMV) enhancer-MCS-3FLAG-SV40-Puromycin vector (Supplementary Fig. 3B) provided by Shanghai Genechem, Inc. An empty vector plasmid was used as a control. The recombinant MAT2A expression plasmids and corresponding control vectors (final concentration of plasmids: 1.65 μg/mL) were transfected into HT22 cells after METTL16 knockdown using Lipofectamine 3000 (#L3000015, Thermo Fisher Scientific). The cells were maintained in culture for 2–4 days, and the overexpression efficiency was determined using western blotting and qRT–PCR as described above.

To validate the effect of the METTL16 methylation site of MAT2A-mRNA-3′-UTR on the stability of MAT2A mRNA, the wild CDS-3′-UTR or mutant CDS-3′-UTR sequences of MAT2A mRNA was inserted between BamHI and HindIII of the CMV enhancer-MCS-3FLAG-SV40-Puromycin vector (Supplementary Fig. 3B) provided by Shanghai Genechem, Inc. The recombinant wild-MAT2A-3′-UTR and mutant-MAT2A-3′-UTR expression plasmids (final concentration of plasmids: 1.65 μg/mL) were transfected into HT22 cells using Lipofectamine 3000 (#L3000015, Thermo Fisher Scientific) and cultured for another 2–4 days to test the stability of MAT2A mRNA. Table 3 displays the sequence of the wild-type and mutant-MAT2A mRNA-3′-UTR.

**Table 3:** The sequence of wild and mutant-MAT2A mRNA-3′UTR.

Name	Sequence^a^
MAT2A mRNA-3′UTR-wild (2981–3005)	5′ -TGGTGTGGTAC**A**GAGAAGCCAGCTT- 3′
MAT2A mRNA-3′UTR-mut (2981–3005)	5′ -TGGTGTGGTAC**T**GAGAAGCCAGCTT- 3′

^a^The METTL16 methylation site is underlined, and the mutation sequence is bold.

### Virus stereotaxic injections

To knockdown METTL16, Sh-METTL16-3 was packed into the site between two BsmBI of the U6-MCS-CAG-EGFP adeno-associated virus (AAV) vector (Supplementary Fig. 3C; Genechem Inc.), which was named AAV-sh-METTL16. The no-load shRNA AAV, named AAV-control, was used as a control. The viral titre was 1.0 × 10^10^ v.g/μL. Subsequently, 0.5 μL AAV-sh-METTL16 and 0.5 μL AAV-control were delivered specifically to the hippocampal tissues, as described previously [45].

To overexpress MAT2A, the CDS sequence of MAT2A mRNA was inserted between KpnI and BamHI of the CMV bGlobin-MCS-mCherry-3FLAG-WPRE-hGH polyA AAV vector (Supplementary Fig. 3D, Genechem Inc.), and was named AAV-OE-MAT2A. The no-load overexpressing AAV named AAV-control (ctrl) was used as the control. Subsequently, equal volumes of AAV-OE-MAT2A and AAV-sh-METTL16 were mixed and designated as AAV-sh-M16 + OE-MAT2A, while equal volumes of AAV-ctrl and AAV-sh-METTL16 were mixed and designated as AAV-sh-M16 + ctrl. Further, 1 μL AAV-sh-M16 + OE-MAT2A and 1 μL AAV-sh-M16 + ctrl were delivered specifically to the hippocampal tissues, as described previously [45].

After the weight of mice had recovered to the preoperative period and the AAV virus was stable expression (approximately four weeks), behavioural experiments were performed. Subsequently, positive EGFP- and mCherry-fluorescent clones were observed using a fluorescence microscope to examine the expression of EGFP and mCherry for evaluating the accuracy of the microinjection site, and data obtained with inaccurate microinjection site were dropped from the data analysis.

### Y-maze test

Y-maze, as a labyrinth, is composed of three arms of equal length, that is, arms I, II, and III, as well as their junction area. Each arm is 30 cm long, 8 cm wide, and 15 cm high. The Y-maze test was conducted four weeks (28 days) after administering virus stereotaxic injections and was conducted between 08:00 a.m. and 04:00 p.m. The three arms were, respectively, labelled as start arm, new arm, and old arm. The Y-maze test was divided into two phases: training and testing. During the training phase, the new arm was closed, each mouse placed into the start arm was allowed to freely explore the maze for 5 min. After that, the mice were transferred into their home cages for 4 h. During the test phase, the new arm was opened and each mouse was allowed to explore all three arms of the maze for 5 min. After each session, the maze was cleaned with 75% ethanol to eliminate odours between mice. The entries into the new arm, time in the new arm, latency first entrance to the new arm, and total distance of mice were documented and analysed on a Smart v3.0 System (Panlab Inc., Spain).

### Open-field test

After the Y-maze test, the mice were acclimatised for two days to the experimental environment. For open-field test, each mouse was placed in the middle of one of the side walls (facing the wall) in an open-field arena (50 × 50 cm) with opaque walls (40 cm high) between 08:00 a.m. and 04:00 p.m. The spontaneous activity of each mouse was tracked for 5 min. After each session, the field site was cleaned with 75% ethanol to eliminate odours between mice. The total distance, mean speed in the centre, latency first entrance to the centre, and time in the centre were documented and analysed on a Smart v3.0 System (Panlab Inc., Spain).

### Object recognition test (ORT)

ORT relies on rodent natural proclivity for novelty and is employed to assess learning and memory after the open-field test [46]. The ORT was and divided into three phases: training, 2 h test, and 24 h test, and performed between 08:00 a.m. and 04:00 p.m. During the training phase, each mouse was given 5 min to explore the environment with two identical objects. Subsequently, one of the familiar objects was replaced with a novel one. During the 2 h and 24 h test phase, each mouse was placed in the apparatus and given 5 min to explore the two distinct objects. The Discrimination index (DI) during the test phase was calculated as:

$${\mathrm{DI}} = \frac{{{\mathrm{TN}}}}{{({\mathrm{TF}} + {\mathrm{TN}})}}$$

where, TN is the time exploring novel object, and TF is the time exploring familiar object.

### MWM test

After ORT, spatial learning and memory were tested by MWM test as described by our previous publication [45]. The MWM test was performed between 08:00 a.m. and 04:00 p.m. and divided into three phases: visible platform, training trial, and probe trial. In the visible platform phase, the escape platform surface (10 cm in diameter) was 1 cm above the surface of the water, and the platform was moved randomly between four locations in four trials. Afterward, the platform was submerged during the training trial phase. The training paradigm in this phase consisted of 5 consecutive test days with four trials per day. Each trial began with mice from one of the four quadrants with their backs to the wall of the pool and ended when they climbed onto the escape platform. A trial terminated when the mice climbed onto the platform and remained on it for at least 5 s. If the mice did not climb onto the platform in 60 s, they would guide to the platform. After the training, the underwater platform was removed from the pool during the probe trial phase. All swimming trajectories of mice were systematically recorded, and the average velocity (visible platform phase), latency to find the platform (visible platform and training trial phases), number of platform crossings (probe trial phase), and time in target quadrants (probe trial phase) were analysed using a Smart v3.0 System (Panlab Inc., Spain).

### Golgi-Cox staining

After the MWM test, the mice were anaesthetised using 1% pentobarbital sodium by intraperitoneal injection (0.1 mL/20 g) and transcardially perfused with 40 mL chilled 0.9% saline solution, then perfused with 40 mL chilled 4% paraformaldehyde. After perfusion, the brains were excised immediately and fixed in 4% paraformaldehyde for 24 h prior to being embedded in Golgi staining solution. Golgi staining was performed in accordance with the protocol provided by the neurone Golgi staining kit (#GMS80020.1, Genmed Scientifics Inc., USA). Brains were cut in 100-μm sections by using a vibratome (#VT100S, Leica, Germany). Dendritic spines on the secondary branches of apical and basal dendrites in the hippocampal area were used for imaging using a microscope equipped with a ×100 oil immersion objective lens (#DM2000 LED, Leica Germany). Quantification was performed in a blinded fashion. In brief, only pyramidal neurones that appeared to be fully impregnated, not obscured by neighbouring pyramidal neurones, and had no apparent truncation of their dendritic profiles were utilised and analysed. Further, six well-impregnated neurones that were clearly isolated from adjacent cells in each brain were randomly selected for analysed. Five representative dendrites of 10 μm (or longer) were randomly chosen per neurone for closer inspection (via ×100 oil immersion lens) to quantify hippocampal spine density. Spines with a neck were labelled as thin/mushroom-like using previously described criteria [47, 48], while those without a marked neck were categorised as stubby. Spine density and quantitative morphological analyses of individual dendritic spines were performed using ImageJ software (National Institutes of Health, Bethesda, MD, USA).

### MAT2A mRNA stability test

For RNA stability analysis, 5 μg/mL actinomycin (ActD, #17505, AAT Bioquest, USA) was added to the cells at 30% confluence after knocking down METTL16 or overexpressing MAT2A mRNA-3′-UTR-wild or MAT2A mRNA-3′-UTR-mut in HT22 cells as described above. Then, the cells were harvested to isolate total RNA at five time points (0, 2, 4, 6, and 8 h) after the treatment of ActD, Finally, RNA quantification for MAT2A mRNA and U1 snRNA was performed using qRT–PCR as mentioned above.

### RNA immunoprecipitation (RIP)-qRT–PCR

HT22 cells were plated in 10 cm culture dishes. The cells were collected at 95% confluence, and dissolved in 1 mL RIP lysis buffer (20 mM Tris-HCl pH 7.5, 150 mM NaCl, 1 mM EDTA, 0.5% NP-40) supplemented with 40 μL phenylmethylsulfonyl fluoride (#P0100, Solarbio, China) and 4 μL RNase inhibitor (#N8080119, Thermo Fisher Scientific), and incubated overnight at 4 °C with magnetic beads bound with 5 μg of rabbit anti-METTL16 (#TA504710, ORIGENE, USA) or rabbit anti-IgG (#AC005, ABclonal, China) antibodies. After IP, the aliquots of sample solution were then detected by western blotting analysis to verify METTL16 binding. The remnant samples were incubated with protein kinase K (#PD101-01-AB, Vazyme, China) for 30 min at 55 °C with shaking to digest protein. RNA was extracted and purified according to the standard protocol of the Total RNApure Kit (#ZP404-1, ZOMANBIO, China). The RNA was dissolved in nuclease-free water (#P119E; Promega Corporation, USA) and RNA quantification of MAT2A mRNA was performed using qRT–PCR, as described above.

### RNA m^6^A dot blotting

RNA m^6^A dot blotting was employed to verify m^6^A binding with anti-m^6^A antibody. HT22 cells were collected at 95% confluence, and total RNA was isolated and purified according to the standard protocol of the Total RNApure Kit (#ZP404-1, ZOMANBIO, China). The 6 μg RNA suspended in 200 μL RIP lysis buffer (20 mM Tris-HCl pH 7.5, 150 mM NaCl, 1 mM EDTA, 0.5% NP-40) supplemented with 0.2 μL RNase inhibitor (#N8080119, Thermo Fisher Scientific), and incubated overnight at 4 °C with magnetic beads bound with 5 μg of rabbit anti-m^6^A (#202003, Synaptic Systems, Germany) or rabbit anti-IgG (#AC005, ABclonal, China) antibodies. After IP, the aliquots of sample solution were then detected by dot blotting analysis to verify m^6^A binding. Briefly, the aliquots above were added to the nitrocellulose membrane (#66485; Biosharp, China). The membrane was then cross-linked by UV light, sealed for 30 min, and incubated overnight at 4 °C in rabbit anti-m^6^A antibody (1:1000, #202003, Synaptic Systems, Germany). After that, the membrane was washed in TBST thrice for 5 min and further incubated for 2 h in corresponding Dylight™ 800 conjugated anti-rabbit IgG (H&L) (GOAT) antibody (1:10,000, #611–145-002, ROCKLAND, USA) at room temperature (~25 °C). After washing thrice again in TBST, the reactive bands were visualised using an Odyssey IR fluorescence scanning imaging system (LICOR, USA).

### Anti-m^6^A RIP-qRT–PCR

After verifying m^6^A binding with the anti-m^6^A antibody, anti-m^6^A RIP-qRT–PCR was used for quantification of m^6^A-modified MAT2A levels. The total RNA was extracted using the total RNApure Kit (#ZP404-1, ZOMANBIO, China) after METTL16 knockdown in HT22 cells. Then 18 μL of total RNA were subjected to fragmentation using Mg^2+^ fragmentation solution (#E6150SNEB, NEB, USA) at 94 °C for 3 min. Subsequently, RNA was purified again using a RNeasy micro kit (#74004, Qiagen, Germany) and quantified using a NanoDrop Lite spectrophotometer (Thermo Fisher Scientific). The 6 μg fragmented RNA suspended in 200 μL RIP lysis buffer (20 mM Tris-HCl pH 7.5, 150 mM NaCl, 1 mM EDTA, 0.5% NP-40) supplemented with 0.2 μL RNase inhibitor (#N8080119, Thermo Fisher Scientific) was subjected to RNA immunoprecipitation, in which 1 μg of a rabbit anti-m^6^A antibody (#202003, Synaptic Systems, Germany) conjugated with 8 μL of Dynabeads protein A (#10001D, Life technologies, USA) was used. After rotation at 4 °C for 2 h, the magnetic beads were washed five times with the RIP lysis buffer, and the precipitated RNA was eluted in the RIP lysis buffer containing 5 mg/mL m^6^A (#B5993, APExBIO, USA) at 55 °C for 30 min. The transcripts of interest contained in the input and immunoprecipitated RNA were quantified using qRT–PCR, as described above.

### Statistical analysis

SPSS (version 22.0) and GraphPad Prism (version 8.0) were used in this study for statistical analysis of the data. All quantitative variables were presented as mean ± standard error of the mean (SEM), which is included at least three independent experiments with consistent outcomes and representative of a minimum of three biological replicates per experiment. First, the Shapiro–Wilk test was used for normal distribution testing. A Student’s unpaired two-tailed *t*-test was used to compare two groups with different variances, a one-way analysis of variance (ANOVA) was used to compare values between more than two groups, and a two-way repeated-measures ANOVA was used for the comparisons of repeated-measures quantitative variables. Post hoc multiple comparisons were performed with the SNK-q test. Statistical significance was established at *P* < 0.05 and indicated by asterisks (*P < 0.05 and **P < 0.01). Details of the statistical analyses, including sample numbers (n), are included in the respective figure legends.

## Supplementary information

Supplementary legends

Supplementary figure 1

Supplementary figure 2

Supplementary figure 3

Supplementary table 1

The mass spectrometry proteomics data

## Data Availability

The mass spectrometry proteomics data were deposited in the ProteomeXchange Consortium (http://proteomecentral.proteomexchange.org) via the iProX partner repository with the dataset identifier PXD032022. Other data generated or analysed during this study are included in the main text and supplementary information files. The datasets used and/or analysed during the current study are available from the corresponding author upon reasonable request.
