# Supplementary material for: Knockdown of METTL16 disrupts learning and memory by reducing the stability of MAT2A mRNA
Source: Cell Death Discov. 2022 Oct 28;8:432. doi: 10.1038/s41420-022-01220-0 (PMC9616879; doi:10.1038/s41420-022-01220-0)
Supplement: Supplementary file 1 — Supplementary legends [file 41420_2022_1220_MOESM1_ESM.docx]

**ADDITIONAL INFORMATION**

**Supplementary information**

**Supplementary Fig. 1. Overexpression of MAT2A in the hippocampi of METTL16 knockdown mice did not affect the emotional state of mice as assessed using an open field test. A–D,** Total distance (**A**), mean speed in centre (**B**), latency 1^st^ entrance to centre (**C**), and time in centre (**D**) of mice in the AAV-sh-M16 + ctrl (n = 9) and AAV-sh-M16 + OE-MAT2A groups (n = 9). Data are expressed as the mean ± standard error of the mean. *P* values were determined using a two-tailed t-test.

**Supplementary Fig. 2. Knockdown of METTL16 in the hippocampi did not affect the emotional state of mice as assessed using an open field test. A–D,** Total distance (**A**), mean speed in centre (**B**), latency 1^st^ entrance to centre (**C**), and time in centre (**D**) of mice in the AAV-control (n = 9) and AAV-sh-METTL16 groups (n = 9). Data are expressed as the mean ± standard error of the mean. *P* values were determined using a two-tailed t-test.

**Supplementary Fig. 3. Empty plasmid map.** The empty plasmid map of hU6-MCS-CBh-gcGFP-IRES-puromycin lentiviruses, which was employed to insert the shRNAs targeting METTL16 **(A)**. The empty plasmid map of cytomegalovirus (CMV) enhancer-MCS-3FLAG-SV40-Puromycin vector, which was employed to construct the MAT2A overexpression plasmid (**B)**. The empty plasmid map of a U6-MCS-CAG-EGFP adeno-associated virus (AAV) vector, which was employed to insert the shRNAs targeting METTL16 (**C)**. The empty plasmid map of CMV bGlobin-MCS-mCherry-3FLAG-WPRE-hGH polyA AAV vector, which was employed to construct the MAT2A overexpression AAV vector (**D)**.

**Supplementary** **Table. 1. The identified parameters and instructions used for MASCOT searches.**
