# Supplementary figures and images for: Knockdown of METTL16 disrupts learning and memory by reducing the stability of MAT2A mRNA
Source: Cell Death Discov. 2022 Oct 28;8:432. doi: 10.1038/s41420-022-01220-0 (PMC9616879; doi:10.1038/s41420-022-01220-0)

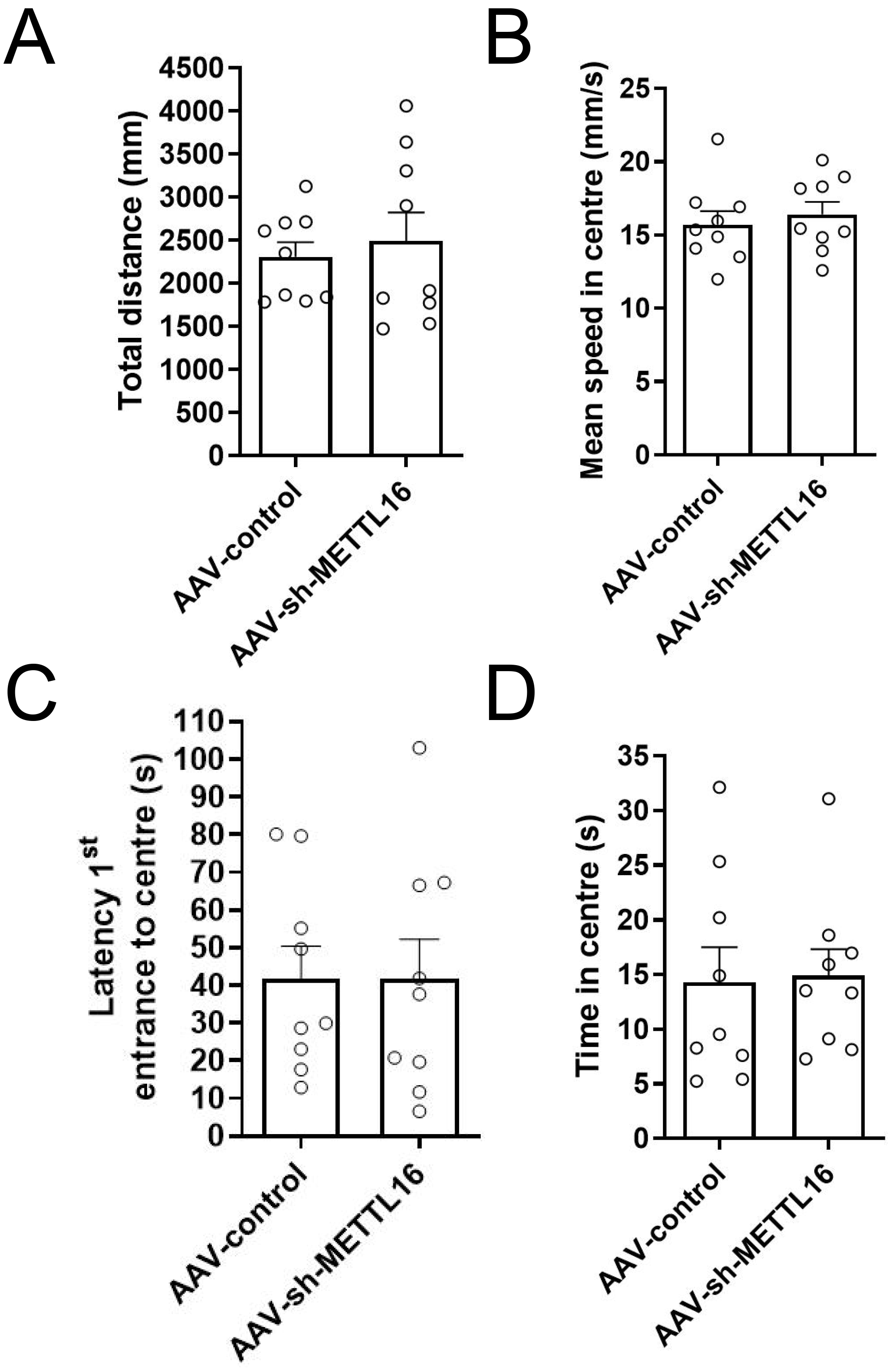

Supplement: Supplementary file 2 — Supplementary figure 1 [file 41420_2022_1220_MOESM2_ESM.jpg]

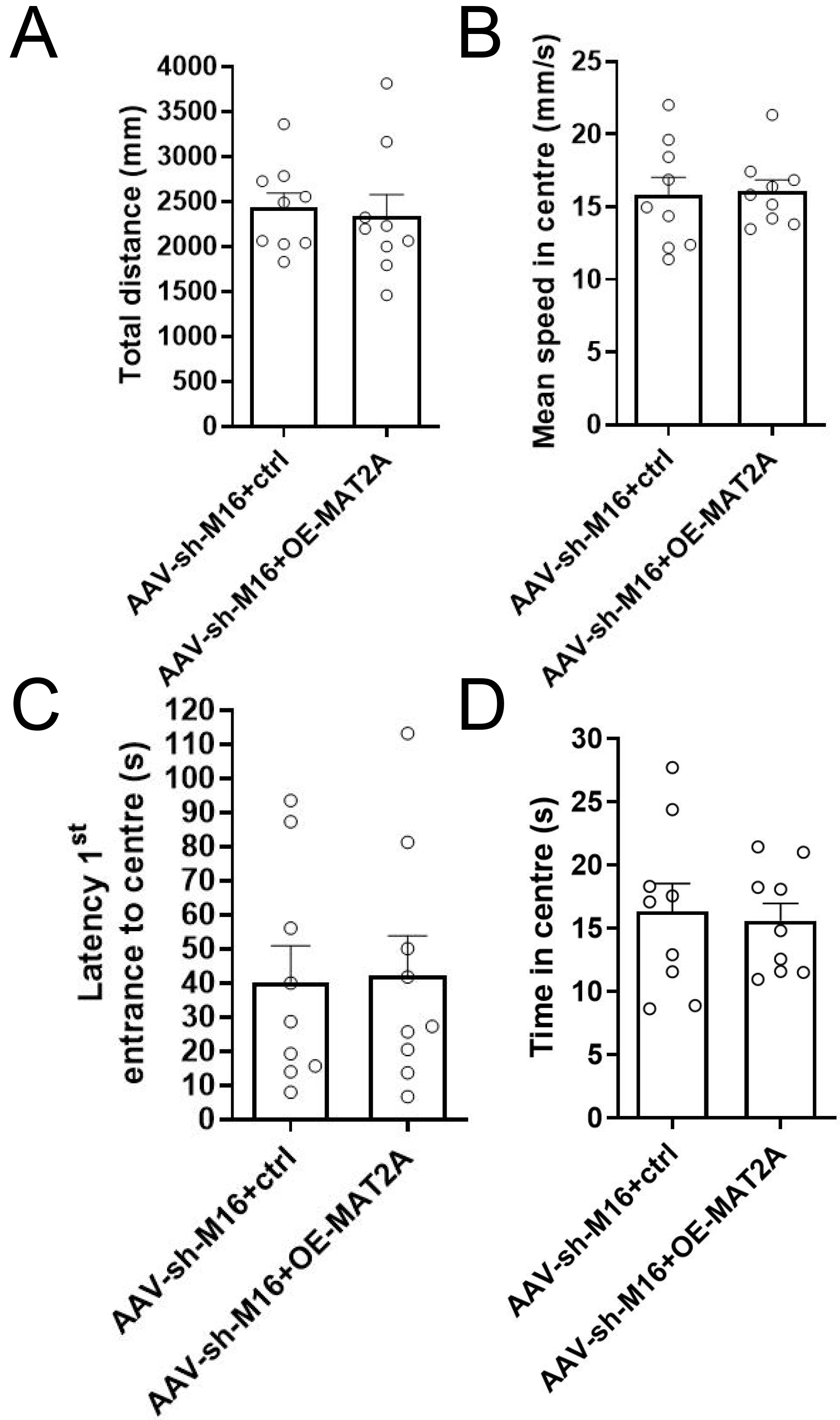

Supplement: Supplementary file 3 — Supplementary figure 2 [file 41420_2022_1220_MOESM3_ESM.jpg]

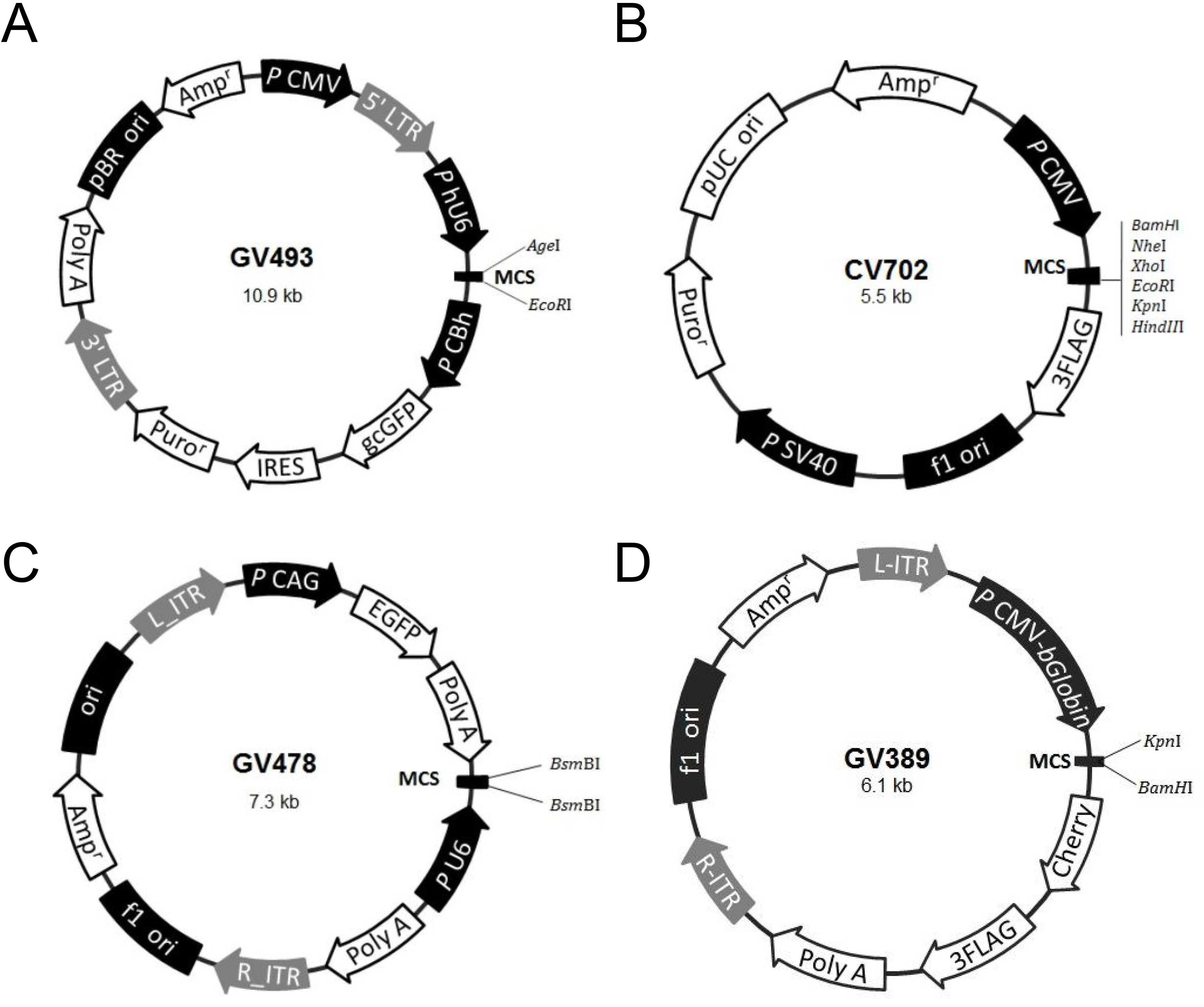

Supplement: Supplementary file 4 — Supplementary figure 3 [file 41420_2022_1220_MOESM4_ESM.jpg]
