# Supplementary material for: Knockdown of METTL16 disrupts learning and memory by reducing the stability of MAT2A mRNA
Source: Cell Death Discov. 2022 Oct 28;8:432. doi: 10.1038/s41420-022-01220-0 (PMC9616879; doi:10.1038/s41420-022-01220-0)
Supplement: Supplementary file 6 — The mass spectrometry proteomics data [file 41420_2022_1220_MOESM6_ESM.docx]

The mass spectrometry proteomics data were deposited in the ProteomeXchange Consortium (<http://proteomecentral.proteomexchange.org>) via the iProX partner repository with the dataset identifier PXD032022.
